# Induction chemotherapy increases efficacy and survival rate of patients with locally advanced esophageal squamous cell carcinoma

**DOI:** 10.3389/fonc.2022.1067838

**Published:** 2022-12-21

**Authors:** Yuting Huang, Jing Chang, Xiaolei Guo, Chao Zhang, Wenping Ji, Shusheng Zhou, Chao Wang, Xu Zhang

**Affiliations:** ^1^ Department of Oncology, Chaohu Hospital of Anhui Medical University, Chaohu, China; ^2^ Department of Oncology, First Affiliated Hospital of Anhui Medical University, Hefei, China; ^3^ Department of Neonatology, Chaohu Hospital of Anhui Medical University, Chaohu, China; ^4^ Department of Scientific Research, Chaohu Hospital of Anhui Medical University, Chaohu, China; ^5^ Department of Epidemiology and Biostatistics, School of Public Health, Anhui Medical University, Hefei, China

**Keywords:** esophageal cancer, concurrent chemoradiotherapy, induction chemotherapy, prognosis, survival

## Abstract

**Objective:**

The efficacy of concurrent chemoradiotherapy (CRT) after induction chemotherapy (IC) in the treatment of esophageal squamous cell carcinoma (ESCC) remains unclear. The purpose of this study was to explore the efficacy of IC in patients with ESCC.

**Methods:**

124 patients with ESCC receiving CRT were included. Patients were divided into IC+CRT group and CRT group. Short-term and long-term efficacy as well as survival time of the two groups were compared, influencing factors of IC efficacy were investigated, and overall survival (OS) and progression-free survival (PFS) between the two groups were compared in different subgroups.

**Results:**

There was no significant difference in the objective response rate (ORR) between the two groups. After IC, the ORR was higher in patients with single-drug concurrent chemotherapy weekly and patients with effective IC. In the long-term efficacy, advanced clinical stage patients had a shorter PFS compared to early-stage patients, and chemoradiotherapy mode ameliorates patients’ PFS. OS and PFS of IC+CRT group were longer than that of CRT group in both tumor diameter <5cm and single-drug chemotherapy weekly subgroups. In addition, OS of IC+CRT group was longer than that of CRT group in pathological grade G1-2 subgroup.

**Conclusions:**

IC improve the efficacy and survival rate of patients with locally advanced ESCC, and the benefits are more advantageous in subgroups of effective IC, pathological grade G1-2, tumor diameter < 5cm, single-drug concurrent chemotherapy weekly.

## 1 Introduction

Esophageal cancer is the sixth most common malignant tumor in the world (1), and its main pathological types are squamous cell carcinoma and adenocarcinoma. China has the highest incidence of esophageal squamous cell carcinoma (ESCC), with 324,000 new cases and 301,000 deaths in 2020 (2). However, in the poor and undeveloped areas of China, most patients have been diagnosed as locally advanced stage at the first visit to a doctor due to the limitation of medical conditions and the lack of regular screening, resulting in low surgical resection rate and low cure rate.

Currently, concurrent radiochemotherapy (CRT) is the first-line treatment for patients with unresectable esophageal cancer (3), but its efficacy is poor. Therefore, increasing clinical trials are actively exploring better treatments for these patients. Some investigators advocate induction chemotherapy (IC) followed by radiotherapy or CRT. The reason is that the tissue and vascular fibrosis after radiotherapy will make it difficult for chemotherapy drugs to invade tumor cells. In addition, CRT may lead to fatal complications (e.g., esophageal tracheal fistula, acute tumor hemorrhage, etc.) for patients with stage IVA esophageal cancer, if they are sensitive to chemoradiotherapy. Therefore, some studies have shown that IC can reduce the risk of complications by shortening patients’ stage. However, these results remain controversial. Previous studies have shown that IC has a survival advantage in patients with stage III, stage IVA, and high-risk factors (4–6).

In this study, we investigated the efficacy and survival rate of patients with locally advanced ESCC in stage III/IVA patients after CRT or IC+CRT treatment, and to further explore the possible beneficiaries of IC.

## 2 Patients and methods

### 2.1 Subjects

A total of 124 patients with ESCC receiving CRT in Chaohu Hospital Affiliated to Anhui Medical University from January 2016 to January 2019 were included in this study according to the 8th edition of American Joint Committee on Cancer/Tumor Node Metastasis (AJCC/TNM) staging system of esophageal cancer (7). Patients’ age, gender, disease stages (i.e., primary tumor (T) and lymph nodes (N)), pathological grade, tumor diameter, and concurrent chemotherapy were recorded and obtained by searching the medical electronic database. All patients had written informed consent, and this study was supported by the Clinical Hospital Research Ethics Committee of the Chaohu Hospital of Anhui Medical University.

### 2.2 Inclusion criteria

Patients with locally advanced unresectable stage III (cT3N1M0, cT1-3N2M0) and stage IVA (cT4N0-2M0, any TN3M0) were included in this study according to the 8th edition of AJCC/TNM staging system of esophageal cancer (7). In addition, patients should meet following criteria: performance status (PS) score was less than 3, imaging had detectable size lesions, patients didn’t have serious organ dysfunction, and routine blood tests and biochemical parameters were normal.

### 2.3 Therapeutic methods

Radiotherapy: 6MV X-ray radiotherapy with elekta Precise linear accelerator was used, and the body position was fixed with thermoplastic film. The radiotherapy range was the planning target volume (PTV), the gross tumor volume (GTV), gross tumor volume of lymph node (GTV_nd), and clinical tumor volume (CTV) formed by the expansion of 5-10mm. The radiotherapy dose was 50-66Gy according to individual tolerance differences, and the fractionated dose was 1.8-2.0 Gy/f.

Chemotherapy: Patients with ESCC received dual-drug three-week or single-drug weekly appropriate during radiotherapy. Chemotherapy drugs included taxol, Cisplatin (DDP), and 5-Fluorouracil (5-FU). IC+CRT group received 2-4 cycles of IC before CRT, and the chemotherapy regimen was taxol+DDP or 5-FU+DDP.

### 2.4 Follow-up

Patients were reviewed 1 month after treatment, then every 3 months for two years, and every 6 months after 2 years. If the disease progresses during the reexamination, patients will be followed-up every 3 months. The last follow-up time was June 2021. Local recurrence was defined as the progression of original esophageal lesions and regional lymph nodes. Distant metastasis was defined as the occurrence of metastasis in new organs or non-regional lymph nodes. Progression-free survival (PFS) was registered according to the last radiographic progress time, and the progress time was obtained from the patient’s history of hospitalization and picture archiving and communication system (PACS) images.

### 2.5 Statistic analysis

Kolmogorov-Smirnov test was used for checking the distribution of continuous data. Skewed data were presented as median with interquartile range (IQR), and their differences were compared by Mann-Whitney U test. Categorical variables were expressed as sample size with percentage, and were compared by pearson chi-square test (or Fishers exact test). Cox regression (univariate analysis) was used to find the potential risk factors of PFS, and then multivariate analysis (automatic selection model) was carried out for variables with P-value less than 0.1 in the univariate analysis. Kaplan-Meier test was used to compare the overall survival (OS) and PFS between the two groups. In addition, subgroup analysis of pathological grade, clinical stage, concurrent chemotherapy regimen, and tumor diameter were conducted, and these results were showed using a forest plot. All statistical analyses were conducted in SPSS 23.0 software (SPSS Inc., Chicago, IL, USA) and diagrams were generated using GraphPad Prism version 5.01 (GraphPad Software, Inc, CA, USA), and P <0.05 was considered statistically significant.

## 3 Result

### 3.1 Demographic and clinical characteristics

A total of 124 patients with ESCC receiving CRT were included, including 62 in IC+CRT group and 62 in CRT group. Median age at diagnosis was 68.0 years, of which 97 (78.2%) were male patients. We found that baseline data (e.g., age, gender, T and N, etc.) of the two groups were balanced and comparable (P>0.05). The follow-up deadline was June 6, 2021, and the median OS time was 18.0 months and PFS was 14.0 months. In addition, IC+CRT group have a longer PFS time (χ^2^= -2.293, P=0.022), OS time (χ^2^= -2.308, P=0.021), and median survival time (χ^2 =^ 6.241, P=0.012) compared to CRT group (**Table 1**).

**Table 1:** Demographic and clinical characteristics of patients with ESCC.

Characteristics	CRT (n=62)	IC+CRT (n=62)	Z/χ^2^	P
Age (years, median (IQR))	69.5 (62.0, 72.0)	67.0 (64.0, 70.0)	-0.969^#^	0.333
Gender (n, %)			0.426^*^	0.514
Male	47 (75.8)	50 (80.6)		
Female	15 (24.2)	12 (19.4)		
T (n, %)			0.824^*^	0.364
T3	38 (61.3)	33 (53.2)		
T4	24 (38.7)	29 (46.8)		
N (n, %)			0.132^*^	0.716
N1-2	35 (56.5)	37 (59.7)		
N3	27 (43.5)	25 (40.3)		
Stage (n, %)			0.035^*^	0.852
III	23 (37.1)	22 (35.5)		
IVA	39 (62.9)	40 (64.5)		
Tumor diameter (n, %)			0.576^*^	0.448
<5cm	43 (69.4)	39 (62.9)		
≥5cm	19 (30.6)	23 (37.1)		
Pathological grade (n, %)			0.035^*^	0.852
G1-G2	40 (64.5)	39 (62.9)		
G3	22 (35.5)	23 (37.1)		
PS score (n, %)			0.890^*^	0.345
0	24 (38.7)	19 (30.6)		
1	38 (61.3)	43 (69.4)		
Concurrent chemotherapy regimen (n, %)			0.304^*^	0.582
Single-drug weekly	39 (62.9)	36 (58.1)		
Dual-drug three-week	23 (37.1)	26 (41.9)		
PFS (month,median (IQR))	11.0 (5.0, 24.3)	16.0 (8.8, 30.3)	-2.293^#^	0.022
OS (month,median (IQR))	15.0 (7.0, 30.3)	21.0 (12.8, 36.3)	-2.308^#^	0.021
Survival time (month, median (95%CI))	15.0 (11.2, 18.8)	24.0 (17.8, 30.2)	6.241^*^	0.012

CRT, Concurrent radiochemotherapy; IQR, Interquartile range; IC, Induction chemotherapy; T, Primary tumor; N, Lymph nodes; OS, Overall survival; PS, Performance status; PFS, Progression free survival.

#, Z value, *, χ^2^ value.

### 3.2 Efficacy evaluation

**Figure 1:**
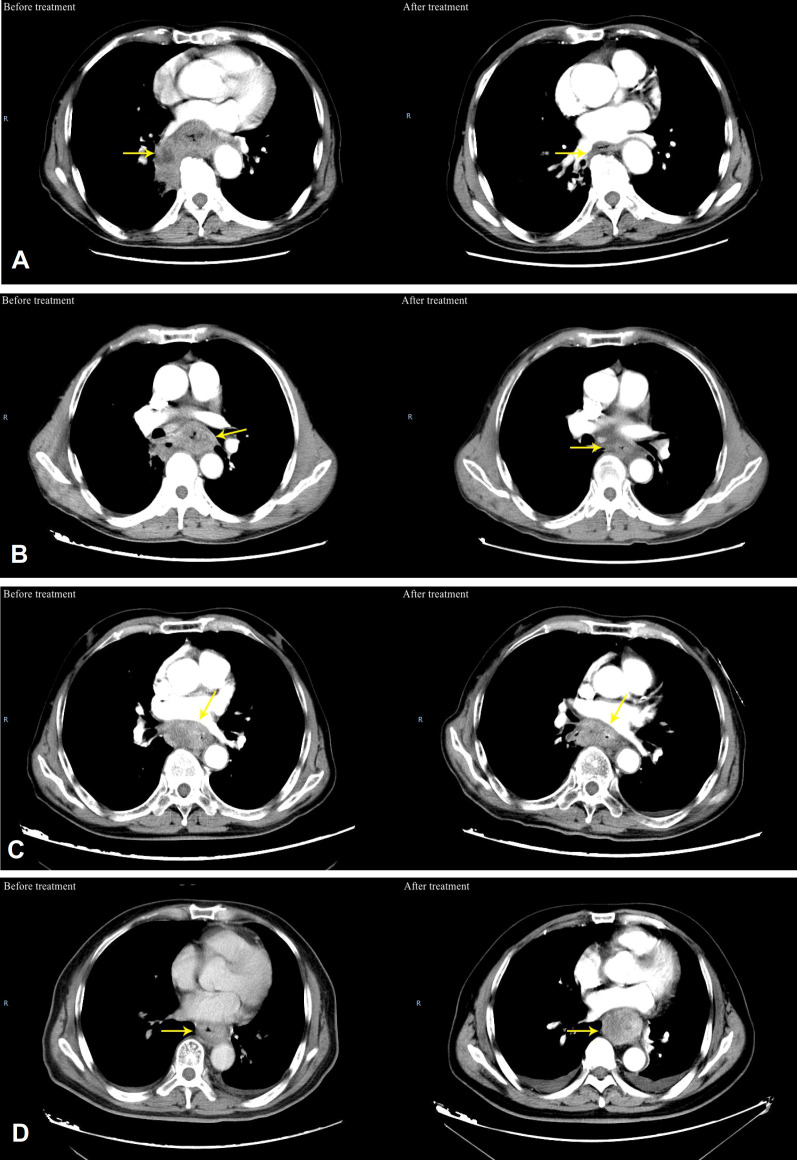
Typical patients of efficacy evaluation before and after treatment for ESCC. **(A)** CR, which means the typical patients of efficacy evaluation before and after treatment for ESCC, **(B)** PR, which means that typical patients of efficacy evaluation before and after treatment for ESCC; **(C)** SD, which means that the sum of esophageal tumor diameters decreased by < 30% on CT after treatment; **(D)** PD which means that the sum of esophageal tumor diameters increased by ≥20% on CT after treatment).

The IC+CRT group evaluated the chemotherapy efficacy 2 weeks after IC, and all patients evaluated the overall efficacy 3-4 weeks after CRT (**Figure 1**). Evaluation methods included esophagography, chestplusabdominal CT, and esophagoscopy. According to Response Evaluation Criteria In Solid Tumors (RECIST1.1) (8), the efficacy of patients after treatment was divided into complete remission (CR), partial remission (PR), stable disease (SD), and progressive disease (PD).

### 3.3 Short-term efficacy in overall patients

Objective response rate (ORR) was 81 (65.3%), including 36 (58.1%) in CRT group and 45 (72.6%) in IC+CRT group. In addition, the ORR of T3 (ORR: 71.8%) and stage III (ORR: 75.6%) were better than that of T4 (ORR:56.6%) and stage IVA (ORR: 59.5%) but without significant differences, respectively (**Table 2**).

**Table 2:** Evaluation of short-term chemotherapy.

Characteristics	Effective (CR+PR)	Non-effective (SD+PD)	*χ^2^*	*P*
Overall	81 (65.3)	43 (34.7)		
Chemoradiotherapy (n, %)			2.884	0.089
CRT	36 (58.1)	26 (41.9)		
IC+CRT	45 (72.6)	17 (27.4)		
Age (years)			1.335	0.248
<65	22 (57.9)	16 (42.1)		
≥65	59 (68.6)	27 (31.4)		
Gender (n, %)			0.028	0.868
Male	63 (64.9)	34 (35.1)		
Female	18 (66.7)	9 (33.3)		
T (n, %)			3.106	0.078
T3	51 (71.8)	20 (28.2)		
T4	30 (56.6)	23 (43.4)		
N (n, %)			0.566	0.452
N1-2	49 (68.1)	23 (31.9)		
N3	32 (61.5)	20 (38.5)		
Clinical stage (n, %)			3.265	0.071
III	34 (75.6)	11 (24.4)		
IVA	47 (59.5)	32 (40.5)		
Tumor diameter (cm, n, %)			0.328	0.567
<5	55 (67.1)	27 (32.9)		
≥5	26 (61.9)	16 (38.1)		
Pathological grade (n, %)			0.056	0.812
G1-G2	51 (64.6)	28 (35.4)		
G3	30 (66.7)	15 (33.3)		
PS score			0.001	0.972
0	28 (65.1)	15 (34.9)		
1	53 (65.4)	28 (34.6)		
Concurrent chemotherapy regimen (n, %)			2.393	0.122
Single-drug weekly	53 (70.7)	22 (29.3)		
Dual-drug three-week	28 (57.1)	21 (42.9)		
Distant metastasis after disease progression (n, %)			0.431	0.511
No	37 (68.5)	17 (31.5)		
Yes	44 (62.9)	26 (37.1)		
Local recurrence after disease progression (n, %)			0.823	0.364
No	37 (69.8)	16 (30.2)		
Yes	44 (62.0)	27 (38.0)		

T, Primary tumor; N, Lymph nodes; PS, Performance status.

### 3.4 Short-term efficacy in IC+CRT group patients

ORR was related to the concurrent chemotherapy regimen (*χ^2 =^* 4.987, *P*=0.026) and the efficacy of IC (Fisher’s exact *P*=0.001) in IC+CRT group. In addition, the ORR of single-drug weekly (83.3%) and effective IC (93.1%) was significantly higher than that of dual-drug three-week (57.7%) and non-effective IC (54.5%), respectively (**Table 3**).

**Table 3:** Evaluation of short-term chemotherapy in the IC+CRT group.

Characteristics	Effective (CR+PR)	Non-effective (SD+PD)	*χ^2^*	*P*
Overall	45 (72.6)	17 (27.4)		
Age (years)			1.677	0.195
<65	11 (61.1)	7 (38.9)		
≥65	34 (77.3)	10 (22.7)		
Gender (n, %)			NA	1
Male	36 (72.0)	14 (28.0)		
Female	9 (75.0)	3 (25.0)		
T (n, %)			0.358	0.550
T3	25 (75.8)	8 (24.2)		
T4	20 (69.0)	9 (31.0)		
N (n, %)			0.246	0.620
N1-2	26 (70.3)	11 (29.7)		
N3	19 (76.0)	6 (24.0)		
Clinical stage (n, %)			NA	0.372
III	18 (81.8)	4 (18.2)		
IVA	27 (67.5)	13 (32.5)		
Tumor diameter (cm, n, %)			2.520	0.112
<5	31 (79.5)	8 (20.5)		
≥5	14 (60.9)	9 (39.1)		
Pathology grade (n, %)			0.332	0.565
G1-G2	30 (75.0)	10 (25.0)		
G3	15 (68.2)	7 (31.8)		
PS score (n, %)			0.238	0.626
0	13 (68.4)	6 (31.6)		
1	32 (74.4)	11 (25.6)		
Concurrent chemotherapy regimen (n, %)			4.987	0.026
Single-drug weekly	30 (83.3)	6 (16.7)		
Dual-drug three-week	15 (57.7)	11 (42.3)		
Distant metastasis after disease progression (n, %)			NA	0.084
No	23 (85.2)	4 (14.8)		
Yes	22 (62.9)	13 (37.1)		
Local recurrence after disease progression (n, %)			0.921	0.337
No	37 (69.8)	16 (30.2)		
Yes	23 (67.6)	11 (32.4)		

T, Primary tumor; N, Lymph nodes; NA, Not available; PS, Performance status.

### 3.5 Risk factors of PFS

Cox univariate regression analysis showed that clinical stage (*HR*=1.737, *P*=0.005) was a risk factor for PFS, i.e., the higher the clinical stage, the shorter the progression-free survival. Furthermore, multivariate analysis showed that clinical stage (*HR*=1.732, *P*=0.033) was a risk factor for PFS, while chemoradiotherapy mode (*HR*=0.628, *P*=0.016) might ameliorate patients’ PFS (**Table 4**). The same findings occurred in OS (Data not shown), i.e., patients with stage IVA had shorter PFS and OS, and patients treated with IC+CRT had longer PFS and OS.

**Table 4:** Risk factors of Progression free survival.

	*HR* (95% CI)	*P*
Univariate analysis		
Chemoradiotherapy (IC+CRT vs. CRT)	0.705 (0.489, 1.018)	0.062
Age	1.048 (0.704, 1.560)	0.816
Gender (Male vs. Female)	1.053 (0.680, 1.633)	0.816
T (T4 vs. T3)	1.352 (0.928, 1.969)	0.116
N (N3 vs. N1-2)	1.424 (0.976, 2.078)	0.067
Clinical stage (IVA vs. III)	1.737 (1.183, 2.549)	0.005
Tumor diameter (≥5cm vs. <5cm)	1.409 (0.943, 2.105)	0.094
Pathological grade (G3 vs. G1-2)	1.165 (0.794, 1.708)	0.434
PS score (1 vs. 0)	1.205 (0.817, 1.776)	0.347
CRT (Twice in three weeks vs. Once a week)	1.195 (0.822, 1.736)	0.351
Multivariate analysis		
Chemoradiotherapy (IC+CRT vs. CRT)	0.628 (0.429, 0.918)	0.016
N (N3 vs. N1-2)	1.216 (0.740, 2.000)	0.440
Clinical stage (IVA vs. III)	1.732 (1.045, 2.870)	0.033
Tumor diameter (≥5cm vs. <5cm)	1.316 (0.864, 2.005)	0.201

T, Primary tumor; N, Lymph nodes; PS, Performance status.

### 3.6 Subgroup analysis of long-term efficacy

**Figure 2:**
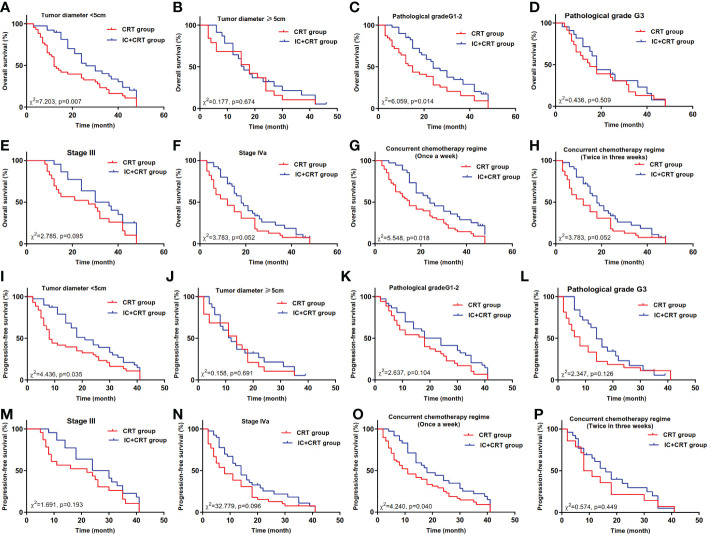
Overall survival and progression-free survival between IC+CRT group and CRT group. **(A–H)**: Overall survival in different subgroups; **(I–P)**: progression-free survival in different subgroups).

In the tumor diameter <5cm subgroup, the median overall survival time (MOST) of IC+CRT group (26 months) was longer than that of CRT group (12 months, *χ^2^=*7.203, *P*=0.007, **Figure 2A**). In the pathological grade G1-2 subgroup, the MOST of IC+CRT group (24 months) was longer than that of CRT group (15 months, *χ^2^=*6.059, *P*=0.014, **Figure 2C**). In the single-drug weekly chemotherapy subgroup, the MOST of IC+CRT group (24 months) was longer than that of CRT group (15 months, *χ^2^=*4.436, *P*=0.035, **Figure 2G**).

**Figure 3:**
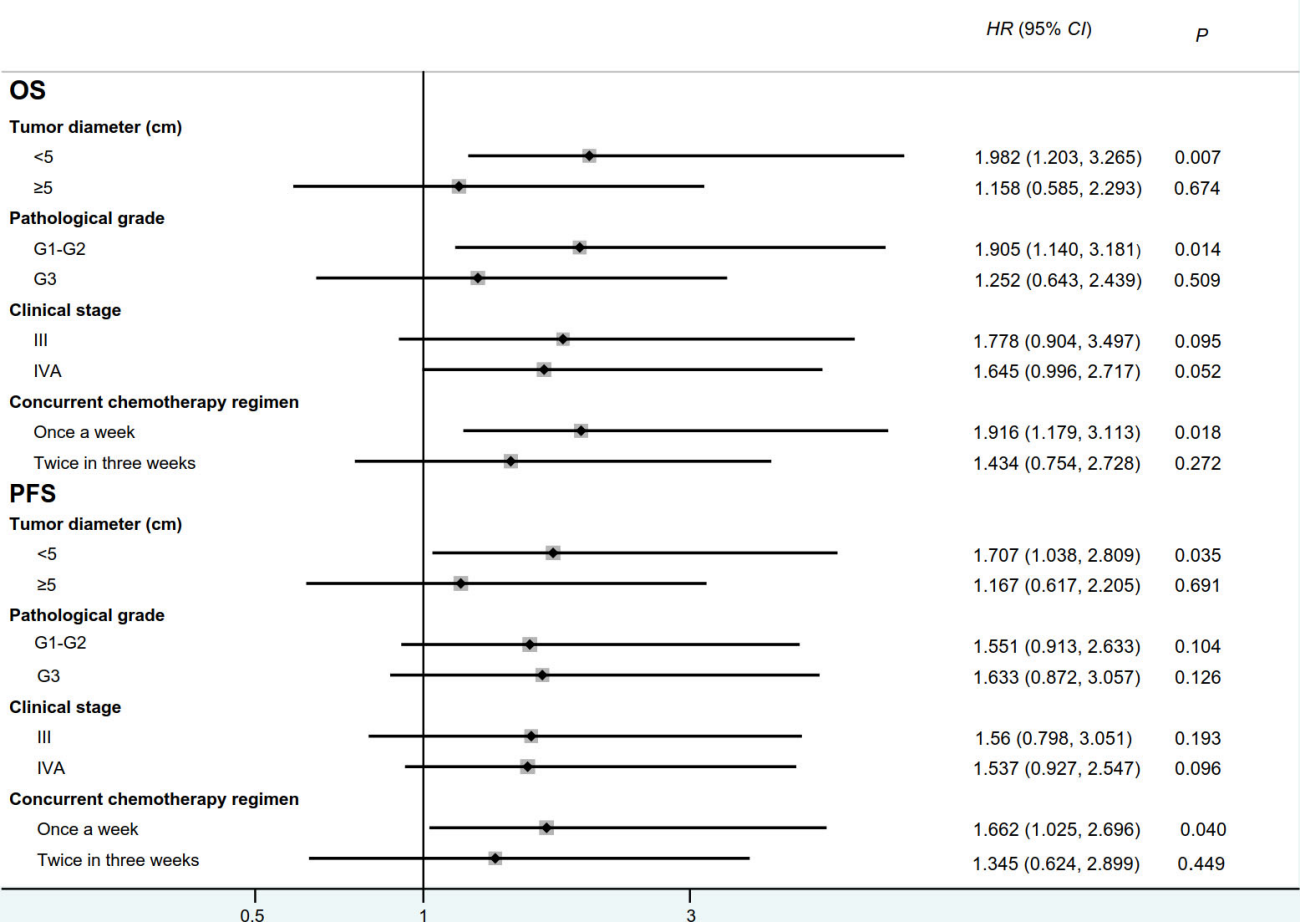
Forest plot for overall survival and progression-free survival between IC+CRT group and CRT group. (OS: overall survival, PFS:progression-free survival; HR:hazard ratio).

In addition, in the tumor diameter <5cm subgroup, the median progression-free survival time (MPFST) of IC+CRT group (21 months) was longer than that of CRT group (8 months, *χ^2^=*4.436, *P*=0.035, **Figure 2I**). In the single-drug weekly chemotherapy subgroup, the MPFST of IC+CRT group (18 months) was longer than that of CRT group (11 months, *χ^2 =^* 4.240, *P*=0.040, **Figure 2O**). There were no significant differences in the remaining comparisons of long-term efficacy (**Figures 2**, **3**).

## 4 Discussion

Induction chemotherapy (IC) was originally used for patients with locally advanced head and neck tumors, and it had become a new standard of neoadjuvant therapy for patients with locally advanced nasopharyngeal carcinoma (9). Currently, there are a few studies on IC for patients with esophageal cancer, and the findings are inconsistent (4, 6, 10). Luo et al. (4) found that the PFS, OS, and 3-year OS rate of patients with esophageal cancer in IC group were higher compared to the non-IC group, but some scholars suggested that IC treatment before CRT treatment could not improve PFS and OS of patients with esophageal cancer. One possible explanation is that these studies have different inclusion criteria for included patients. A prospective study on 110 patients with esophageal cancer who received CRT found that the ORR and OS of IC+CRT group were higher than those of CRT group but without significant difference (5). In addition, subgroup analysis showed that IC was advantageous (with a marginal statistical significance, *P*=0.06) to patients with stage III/IV but not to patients with stage II. In order to verify their findings, similar results occurred in our study, and the included patients were stage III/IVA unresectable ESCC.

In the analysis of potential beneficiaries, some results caught our attention. Firstly, in the subgroup of tumor diameter ≥5cm, IC had no significant survival advantage. In fact, IC was expected for patients with large tumors, and CRT can be performed after tumor shrinkage. These treatments can reduce the size of radiation field, reduce the radiation dose of organs, and increase the radiation dose of tumors. However, we did not observe the advantage of IC in large tumors. The chemotherapy sensitivity of ESCC was poor, and the effective rate of first-line chemotherapy was generally 30-58% (11–13). In the Norton-Simon model (14), gmopertzian growth curve showed that the tumor growth rate will gradually slow down with the growth of the tumor volume, and the chemotherapy efficacy was positively correlated with the tumor growth rate. Therefore, these results suggested that the killing ability of chemotherapy is weak and the effect is poor for patients with large tumors. Their results have shown that 23 patients with tumor diameter ≥5cm chose IC, but only 4 patients achieved PR (ORR was 17.4%). However, small tumor was sensitive to chemotherapy, which will accelerate the tumor reproliferation. In this study, the OS of patients with tumor diameter <5cm was prolonged after IC treatment, which was consistent with the Norton-Simon model (14). Secondly, there was a better prognosis for patients with single-drug chemotherapy, while dual-drug concurrent chemotherapy did not show greater efficacy. Currently, there is no consensus on the combined use and dosage of IC (15). IC can increase the probability of grade III-IV myelosuppression and systemic adverse reactions during radiotherapy (16). In this study, patients who received CRT with dual-drug in three weeks after IC had higher hematological toxicity, radiation reaction, and systemic reactions than other patients. In addition, after receiving dual-drug CRT within 3 weeks, some patients were forced to complete the treatment due to interruption of follow-up synchronous chemotherapy, adjustment of radiotherapy dose and extension of radiotherapy time, resulting in adverse reactions to the prognosis. Finally, in the pathological grade G1-2 subgroup, the prognosis of IC patients was better. Chemotherapy reaction is related to the differentiation degree of tumor cells, i.e., the lower the cell differentiation degree, the faster the tumor proliferation and the higher the chemotherapy reaction. Therefore, it seems more reasonable to choose IC in the G3 subgroup, because IC has higher sensitivity. However, this study failed to observe the advantage of IC in the G3 subgroup patients. Shimodaira et al. (17) included 126 patients with esophageal cancer who underwent surgery after CRT. Of which, 63 patients were treated with IC before CRT. Their results showed that IC was more effective in the G1-2 subgroup patients, and the 5-year OS rate of IC group and non-IC group was 74% and 50%, respectively, but G3 group IC patients had no advantage. NCCTGN0849 (18) also found that IC can improve the OS of esophageal cancer patients in pathological grade G1-2 group, which is consistent with our study. However, the authors did not explore the reasons, further research is needed to explore.

Our research includes several novel findings. Firstly, we found that IC treatment before CRT treatment is more suitable for the treatment of patients with small tumors. Secondly, the toxicity reaction of IC may damage the patient’s body, suggesting that the follow-up CRT should choose single-drug weekly sensitization chemotherapy to obtain a longer survival time.

Some limitation should be considered in this study. Firstly, this is a retrospective study with a limited sample size, thus these results require a larger sample size in different chorots to be verified. Chemotherapy regimens only include single-drug weekly and dual-drug three-week for the treatment may bias our conclusion, although subgroup analyses have been conducted.

In conclusion, IC treatment before CRT can improve the efficacy and survival rate of patients with locally advanced ESCC, especially patients with pathologic stage G1-2, tumor diameter < 5cm, single-drug weekly concurrent chemotherapy, and patients who responded to IC.

## Data availability statement

The original contributions presented in the study are included in the article/Supplementary Material. Further inquiries can be directed to the corresponding authors.

## Ethics statement

Written informed consent was obtained from the individual(s) for the publication of any potentially identifiable images or data included in this article.

## Author contributions

YH, XZ and CW conceived the idea for the study. YH drafted the protocol with input from XZ, XG, JC, and CZ recruited and screened patients. WJ and SZ collected data. YH and XZ analyzed and interpreted data. All authors contributed to the article and approved the submitted version.
